# Supplementary material for: Antifouling Property of Oppositely Charged Titania Nanosheet Assembled on Thin Film Composite Reverse Osmosis Membrane for Highly Concentrated Oily Saline Water Treatment
Source: Membranes (Basel). 2020 Sep 16;10(9):237. doi: 10.3390/membranes10090237 (PMC7558336; doi:10.3390/membranes10090237)
Supplement: Supplementary file 1 [file membranes-10-00237-s001.pdf]

Supplementary Materials

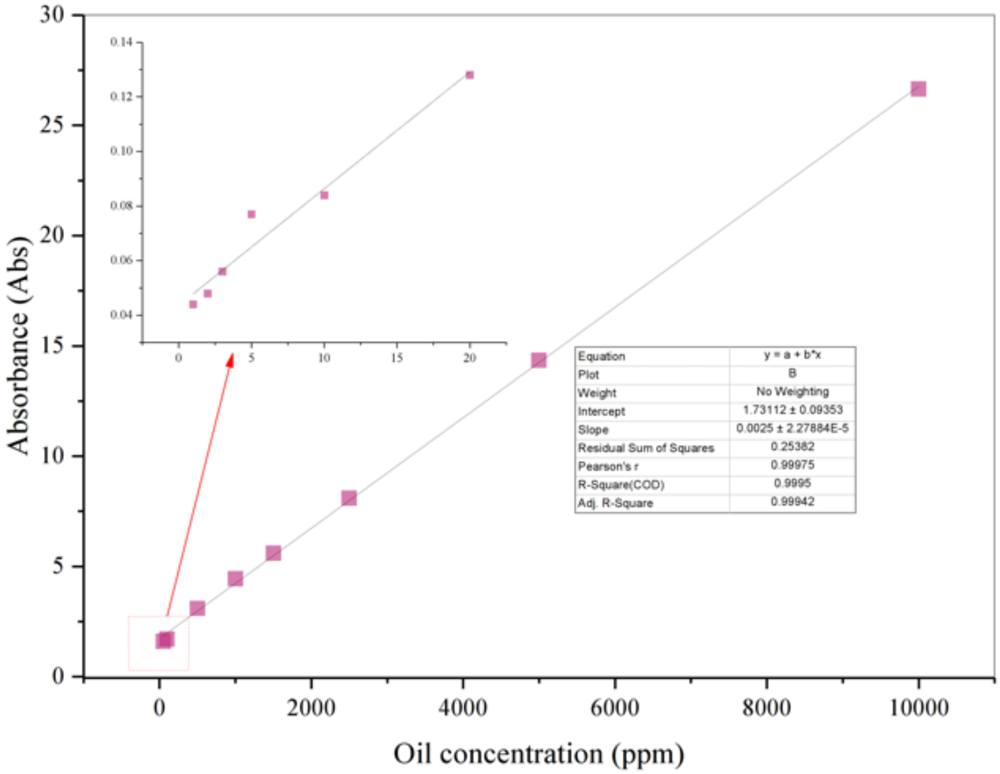

Figure S1 Calibration curve of absorbance with oily saline feedwater.

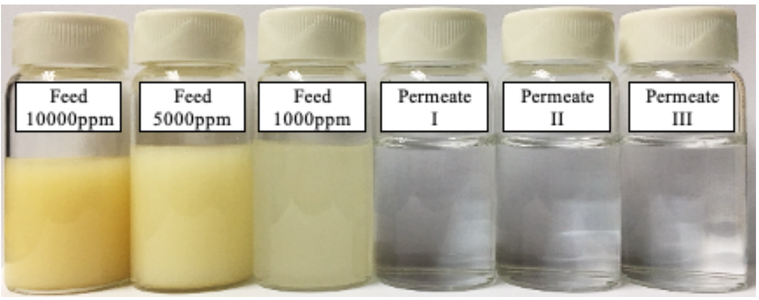

Figure 2S Photographs comparison between the synthetic oily saline wastewater with 1,000ppm, 5,000ppm and 10,000ppm as feedwater and the permeation of each concentration by 2TNS-TFN membrane.
